# Abnormal Accumulation of Collagen Type I Due to the Loss of Discoidin Domain Receptor 2 (Ddr2) Promotes Testicular Interstitial Dysfunction

**DOI:** 10.1371/journal.pone.0131947

**Published:** 2015-07-09

**Authors:** Chu-chao Zhu, Bin Tang, Jin Su, Hu Zhao, Xin Bu, Zhen Li, Jie Zhao, Wei-dong Gong, Zhi-qun Wu, Li-bo Yao, Wei Li, Yuan-qiang Zhang

**Affiliations:** 1 Department of Human Anatomy, Histology and Embryology, Fourth Military Medical University, Xi’an 710032, China; 2 Department of International Medical, China-Japan Friendship Hospital, Beijing 100029, China; 3 Department of Biochemistry and Molecular Biology, State Key Laboratory of Cancer Biology, Fourth Military Medical University, Xi’an 710032, China; 4 Department of Interventional Radiology, Tangdu Hospital, Fourth Military Medical University, Xi’an 710038, China; National Cancer Institute, UNITED STATES

## Abstract

**Background:**

Loss of functional allele for discoidin domain receptor 2 (Ddr2) results in impaired Leydig cell response to luteinizing hormone (LH), low testosterone production and arrested spermatogenesis in older male Ddr2^*slie/slie*^ mice. However, the underlying mechanism responsible for this phenotype remains unknown. Herein, we reported for the first time that the deregulated expression of Ddr2 cognate ligand, namely collagen type I (COL1), may account for the disruption of the testicular steroidogenesis in Ddr2*^slie/slie^* mutant testes.

**Methodology/Principal Findings:**

Expression of Ddr2 increased gradually along postnatal development, whereas COL1 expression became negligible from adulthood onwards. In Ddr2^*slie/slie*^ mutant testis, however, in contrast to the undetectable staining of Ddr2, COL1 expression was constantly detected, with the highest values detected during adulthood. In the experimental vasectomy model, Ddr2^*slie/slie*^ mutant mice exhibited an early androgen deficiency than wild-type mice, along with the accumulation of fibrotic tissue in the interstitium. Functionally, ablation of endogenous Ddr2 resulted in a significant decrease of testosterone (T) level in TM3 cells in the presence of higher concentration of COL1 treatment. Conversely, overexpression of Ddr2 could help TM3 cells to maintain a normal testicular steroidogenesis even in the presence of high concentration of COL1. Additionally, attenuated expression of Ddr2 correlates to the deregulated level of serum T levels in human pathological testes.

**Conclusions:**

Abnormal accumulation of interstitial COL1 may be responsible for the steroidogenic dysfunction in Ddr2^*slie/slie*^ mutant testes.

## Introduction

The discoidin domain receptor superfamily, belonging to the receptor tyrosine kinase (RTK) family, mainly consists of DDR1 and DDR2. Their activations require binding by differential collagens [1]. Several intrinsic characteristics make DDRs different from other RTKs. In stead of growth factors, activation of DDRs requires collagens binding. This activation occurs and maintains within a longer time window [2]. Moreover, even DDR1 and DDR2 have different expression patterns and initiating mechanisms. DDR1 expression is limited to epithelial tissues and its activation needs collagen types I, IV, and V [3]. By contrast, Ddr2 expression is more ubiquitous, and heart, kidney, lung, ovary and testis have all been reported to be positive [4]. Ddr2 is activated mainly by collagen type I (COL1), and other collagens can only exert slight stimulatory effects [2]. Moreover, Ddr1 or Ddr2 knockout mice have different phenotypes [5]. Ddr1 mutant females exhibit reproductive defects. Comparatively speaking, Ddr2 knockout mice suffer a severe disruption of skeletal system regardless of sex difference [6].

Very recently, Kano *et al*. identified a recessive, loss-of-function allele for Ddr2 (designated as Ddr2^*slie/slie*^) that causes dwarfism and infertility of a peripheral origin [7]. Ddr2 is normally expressed in Leydig cells (LCs) of adult wild-type mice. In Ddr2^*slie/slie*^ mice, spermatogenesis almost ceases at 5 months of age. In line with this phenotype, the serum testosterone (T) level, as well as other key steroidogenic factors, was all substantially reduced from postnatal 5 months onwards. It is therefore a logical hypothesis that the disrupted response to LH by LCs, and the collapsed T production may cause the fertility impairment in older Ddr2^*slie/slie*^ mutant testes [8]. Nevertheless, the mechanistic relevance of Ddr2 signaling in such peripheral systems remains to be fully delineated.

In recent years, it has become evident that different factors with key roles in cell proliferation, cell adhesion, and extracellular matrix (ECM) remodeling are potentially involved in the regulation of testicular steroidogenic function [9–15]. The identification of Ddr2 as an essential component in testicular interstitium and the involvement of collagen signaling in the modulation of ECM homeostasis prompted us to evaluate whether the deregulation of Ddr2/COL1 signaling causes the disruption of steroidogenesis in Ddr2^*slie/slie*^ mutant testes. Moreover, deregulation of Ddr2 testicular expression, as well as its correlation with COL1 signaling in human pathological testes was assessed using immunohistochemistry. Our combined analyses are suggestive of a potential action of Ddr2/COL1cascade in LCs, underscoring an unexpected reproductive facet of this newly characterized RTK.

## Materials and Methods

### Human tissue

All patients without obstructive azoospermia have given informed written consent to the procedures and to further study with their redundant testicular tissue material. To be specific, testicular biopsies from the following patients were analyzed: men with hypospermatogenesis (n = 10), and men with spermatogenic arrest at the level of round spermatids (n = 10), as well as Sertoli cell only syndrome (n = 8). In addition, testicular tissues from normozoospermic patients who underwent testicular biopsy during genital surgery procedures for varicocele or epididymal cysts (n = 8) were also included as normal control after identification of normal spermatogenesis status in accordance with the criteria described by Suarez-Quian *et al* [16]. Tissues were immediately fixed in Bouin's fixative after collection and were then processed for routine paraffin embedding. The use of the human tissue in this study was approved by the Human Research Committee of Fourth Military Medical University for Approval of Research Involving Human Subjects.

### Human hormonal profile

The blood samples were collected and processed as described previously [17]. Serum testosterone (T) level was assessed by radioimmunoassay (Diagnostic System Laboratories, Webster, TX, USA) in blood samples obtained between 9 and 10 a.m. All samples were assayed in duplicate. Intra- and inter-assay coefficients of variation (CV) were approximately 5.16 and 6.73% for T.

### Mouse models

Eight-week-old outbred male C57BL/6 mice were obtained from the Laboratory Animal Center of Fourth Military Medical University. The day the litters were born was considered day (d) 1 of age. Mice were housed at 22°C and a light: dark cycle of 12: 12 hours and were weaned at d 21 of age in groups of five mice with free access to pelleted food and tap water. At postnatal 35 d, 42 d, 49 d, 70 d, and 5 month, mice were sacrificed under diethyl ether anesthesia, followed by cervical dislocation. The Ddr2^*slie/slie*^ mutant mice in C57BL/6 background were kindly provided by the laboratory of Libo Yao (Department of Biochemistry and Molecular Biology, State Key Laboratory of Cancer Biology, Fourth Military Medical University, Xi’an, China). The experimental vasectomy operation was performed under sodium pentobarbital (50 mg/kg body weight, i.p) anesthesia via a lower midabdominal incision as described elsewhere [18]. Briefly, the vas deferens on each side was exposed without causing injury to the adherent blood vessels and a double ligature thread. The portion of the vas between the two ligatures was removed and the ligated ends were replaced in the abdominal cavity; the incision was then closed in two layers. The procedure without the vasa deferentia being ligatured was performed as a sham control. At Post-operation 3 and 5 month, mice were killed as described above. For histological studies, some testes were fixed in Bouin’s solution for 24 h, embedded in paraffin, and processed into 5-μm-thick sections. Other testes were immediately stored at -80°C for subsequent biochemical analysis. The Ethics Committee for Animal Experiments of the Fourth Military Medical University approved all animal work and the experimental protocols strictly complied with the institutional guidelines and the criteria outlined in the “*Guide for Care and Use of Laboratory Animals*”. All efforts were made to minimize animal suffering.

### Cells treatment

TM3 LCs were infected with siRNA against Ddr2 (Gene-Pharma Co., Ltd, Shanghai, China) or adenovirus expressing Ddr2 (Vector Gene Technology Company, Beijing, China) for 24 h. The siRNA sequences were mouse *Ddr2*-1573 (sense: 5’-GUCCAACUCUACUUAUGAUTT-3’, antisense: 5’-AUCAUAAGUAGAGUUGGACTT-3’) and mouse *Ddr2*-2146 (sense: 5’-GGAGAAUGGAGAUCUUAAUTT-3’, antisense: 5’-AUUAAGAUCUCCAUUCUCCTT-3’); non-silencing control sense: 5’-UUCUCCGAACGUGUCACGUTT-3’; antisense: 5’-ACGUGACACGUUCGGAGAATT-3’. The infection efficiency of adenovirus in TM3 LCs reached higher than 90% while the knock-down efficiency reached lower than 40% as detected using EGFP fluorescence and Western blotting.

For COL1 stimulation assays, TM3 LCs (10^5^/cm^2^) were seeded on 6-well plates uncoated or coated with different concentrations of COL1 (Roche, Germany) range from 6 μg/cm^2^ to 96 μg/cm^2^. To obtain the coated plates we incubated them with COL1for 3 hours at 37°C and the plates were then incubated with 1% BSA for an additional 2 hour. Subsequently, plates were washed with medium, and cells were seeded and incubated for another 24 hours. At the end of the incubation, the supernatants and cells were collected and stored at -70°C.

### *In vitro* radioimmunoassay

TM3 cells were then treated as indicated above, and the T content of medium recovered from each well was determined using a radioimmunoassay kit (Diagnostic System Laboratories, Webster, TX). Intra- and interassay coefficients of variation (CV) were 8.1 and 7.6% for testosterone measurement. All samples were assayed in duplicate, and each experimental data point consisted of three to six samples.

### Real-time PCR

Total RNA was extracted from frozen mouse testis using RNeasy Mini Kit (QIAGEN Inc., Valencia, CA, USA) according to the manufacture’s instructions. For reverse transcription-polymerase chain reaction (RT-PCR), first-strand cDNA was synthesized with Superscript Ⅲ (Rnase H-Reverse Transcriptase; Invitrogen), exactly according to the manufacturer’s instructions and PCR was set up according to Promega's reverse transcription system protocol. The primers used in this study have been reported previously [19]. Amplification of *Gapdh* mRNAs were served as internal controls. All PCR reactions for all samples were repeated at least three times. PCR products were then quantified by SYBR green intercalation using the MiniOpticon system (Bio-Rad Laboratories, Inc., Hercules, CA, USA). *Gapdh* was used to obtain the △△Ct values for the calculation of fold increases.

### Western blotting

Tissues were homogenized in ice-cold RIPA buffer (Tris-HCl 50 mM, NaCl 150 mM, Triton X-100 1% vol/vol, sodium deoxycholate 1% wt/vol, and SDS 0.1% wt/vol pH 7.5) supplemented with complete proteinase-inhibitor cocktail tablets (Roche Diagnostic, Mannheim, Germany). For western blot analysis, 30 μg mouse testicular protein was separated on 8–10% SDS/PAGE and transferred to nitrocellulose membrane (Millipore, Bedford, MA, USA). Membranes were then incubated with primary antibodies including anti-Ddr2 (R&D Systerms, dilution 1:1000), anti-β-actin (Santa Cruz biotechnology, dilution 1:2000) and anti-COL1 (Abcam, dilution 1:1000) in blocking solution overnight at 4°C. After washing with PBS containing 0.05% Tween-20 (PBST), the membranes were incubated with peroxidase conjugated goat anti-rabbit IgG or goat anti-mouse IgG (diluted at 1:10000) for 60 min. The bands were finally detected by using an ECL kit (Amersham Biosciences, Buckinghamshire, UK) according to the manufacturer's instructions.

### Immunohistochemistry

The streptavidin-biotin complex immunohistochemical method was employed in the immunohistochemical assay on serial 5-μm sections as previously described [20]. Briefly, after endogenous peroxidase activity was blocked with 0.5% H_2_O_2_ in methanol for 30 min, slides were incubated with the anti-Ddr2 mouse antibody (1:100 dilutions; R&D Systerms) and anti-COL1 (Abcam, dilution 1:100) diluted in PBS at 4°C overnight in a moist box. Biotinylated rabbit anti-mouse IgG (1:500 dilution; Vectoy) and goat anti-rabbit IgG (1:500 dilution; Vector) was incubated for 1 hr at RT and detected with streptavidin–peroxidase complex. Peroxidases were detected with 0.7 mg/mL 3–3'-diaminobenzidine tetrahydrochloride (Sigma) in 1.6 mg/mL urea hydrogen peroxide, 60 mmol/L Tris buffer, pH 7.6, RT as the chromogen and the sections were briefly counter-stained with hematoxylin. Negative control slides were incubated either with pre-absorbing serum or with PBS substituted for the primary antibody. IHC reactions for all samples were repeated at least three times, and typical results were illustrated. Ddr2 and COL1 protein staining was analyzed microscopically (Axioskop, Zeiss, Oberkochen, Germany) with ×25 or ×40 objectives.

### Immunofluorescence

The immunofluorescent staining of 4% paraformaldehyde-fixed cells or of tissue sections was carried out according to our previous work [21]. Briefly, samples were blocked with 2% sheep and horse sera in PBS for 30 min at room temperature in order to reduce nonspecific background. The sections were then incubated with the blocking solution containing the primary antibody, at 4°C overnight in a moist box. Slides were washed three times in PBS for 10 min prior to addition of rhodamine-labeled antibodies (Jackson Immune Research Laboratories, West Grove, PA, USA). Nuclei were visualized by 10-min staining of 40,6-diamidino-2-phenylindole (DAPI; dilution 1: 2000; Sigma). The sections were mounted in 80% glycerol and examined with an inverted microscope (Axio Imager M1 microscope; Zeiss). The relative DDR2 fluorescence intensity was analyzed using the Image J software, a publically available Java-based image processing program developed by the National Institutes of Health (http://rsbweb.nih.gov/ij/).

### Masson's Trichrome Staining

Testicular sections were deparaffinized, rehydrated and then washed in distilled water for 5 minutes. After being stained in Weigert's iron hematoxylin working solution for 10 minutes, sections were stained in Biebrich scarlet-acid fuchsin solution for another 10 minutes. Subsequently, sections were differentiated in phosphomolybdic-phosphotungstic acid solution for 10 minutes until collagen did not display red. After that sections were transferred directly (without rinse) to aniline blue solution, stained for 5 minutes. Next, sections were rinsed briefly in distilled water and differentiated in 1% acetic acid solution for 2 minutes. Finally, sections were dehydrated very quickly, and were cleared in xylene and mounted with resinous mounting medium.

### Statistical analysis

Experiments were repeated at least three times, and one representative from at least three similar results was presented. Correlation of relative Ddr2 or COL1 immunoreactive content in human testis to serum T level was determined based on *Pearson’s* correlation coefficient, with the aid of SPSS 15.0 software. Quantitative data are presented as mean ± SEM. Results were analyzed for statistically significant differences using analysis of variance, followed by Tukey’s test. Differences were considered significant at *P* < 0.05.

## Results

### Testicular Expression of Ddr2 and COL1 along postnatal development

**Fig 1:**
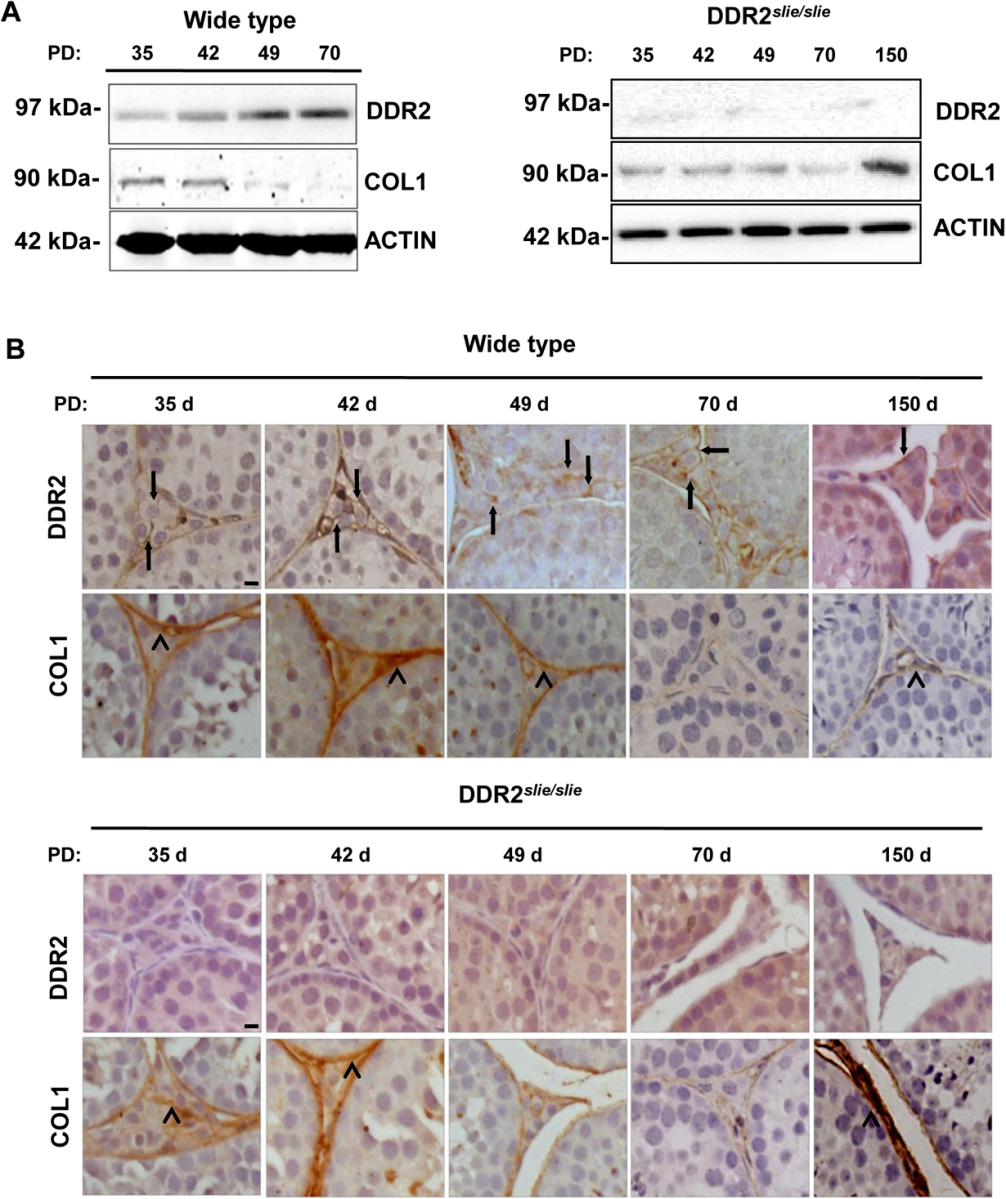
Analysis of DDR2 and COL1 expression in wild-type and Ddr2^*slie/slie*^ mutant testes along postnatal development. **A** Assessment of DDR2 and COL1 protein expression in wild-type and Ddr2^***slie/slie***^ mutant developing testes by western blotting analyses. Immunolabeling of Actin is shown as the loading control. **B** Immunolocalization of DDR2 and COL1 protein in developing wild-type and Ddr2^***slie/slie***^ mutant testes. The arrows indicate DDR2 in LCs and the arrow heads indicate COL1 in testicular interstitium. Bar = 25 μm

**Fig 2:**
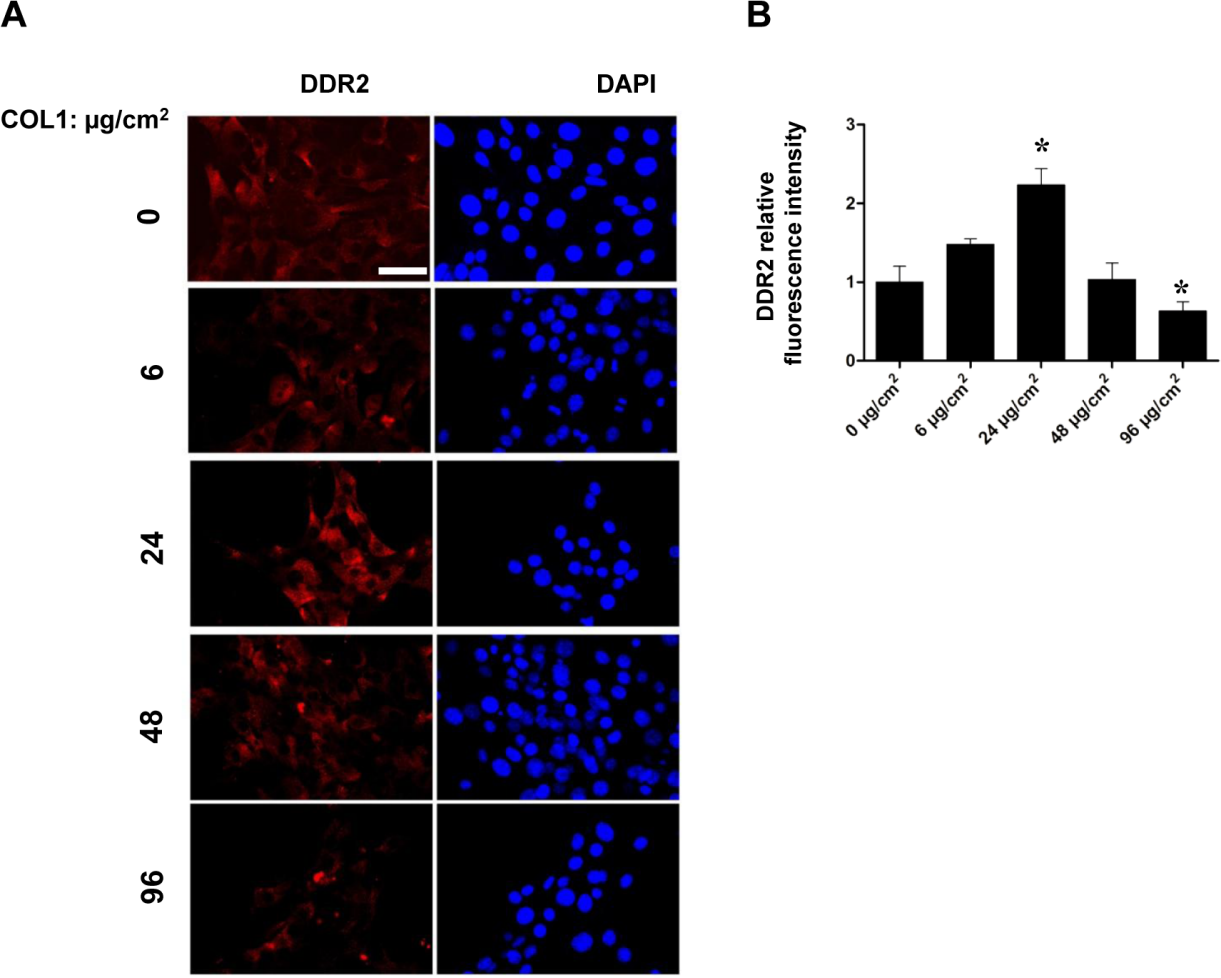
*In vitro* effects of collagen accumulation on DDR2 expression in TM3 cells. **A** Effects of different concentrations of COL1 on DDR2 expression in TM3 cells was illustrated using immunofluorescence. Nuclear were demonstrated using DAPI staining. Bar = 10 μm. **B** Relative fluorescence intensity of DDR2 in panel A was analyzed using Image J. *P < 0 .05 *vs* 0 μg/cm^***2***^ group.

Testicular expression of DDR2 and COL1, at the translational levels, was first explored in wild-type and mutant mouse samples throughout postnatal development. Initial western blotting analyses revealed that the expression levels of DDR2 increased gradually along the study period, with the highest values being detected during the adult period (Postnatal 70 d). In contrast, our immunoblotting analysis showed a declined expression of COL1 in mouse testes during development, with the negligible values in adult (Postnatal 70 d) testis tissue (Fig 1A). In mutant testes, the expression level of COL1 appeared comparable to that of wild-type along the development, with a dramatic increase of COL1 expression in testicular samples from Postnatal 150 d (Fig 1A). Besides expression level analysis, immunolocalization of DDR2 and COL1 in mouse testicular tissue was conducted by means of immunohistochemistry. In wild-type testis, the presence of Ddr2 immunoreactivity was mainly accumulated in the membrane of LCs (arrows in Fig 1B). As for COL1, positive immunoreactivity was mainly mapped to peritubular myoid cells surrounding the seminiferous tubules (arrow heads in Fig 1B). In mutant testis, in contrast to the undetectable staining of DDR2 in LCs of adult testis, expression of COL1 was constantly observed, with the highest values being detected during the adult period (arrow heads in the lower panels of Fig 1B). To further elucidate the deleterious effect of COL1 accumulation on DDR2 expression, we treated TM3 cells with different doses of COL1. It was apparent that DDR2 expression were stimulated by relatively low concentration of COL1 (24 μg/cm^2^), and was significantly inhibited by highest concentration of COL1 (96 μg/cm^2^) in TM3 cells after 24 hours of incubation (Fig 2).

### Susceptivity of Ddr2 slie/slie mutant testis to the experimental-induced testicular interstitial fibrosis by murine vasectomy model

**Fig 3:**
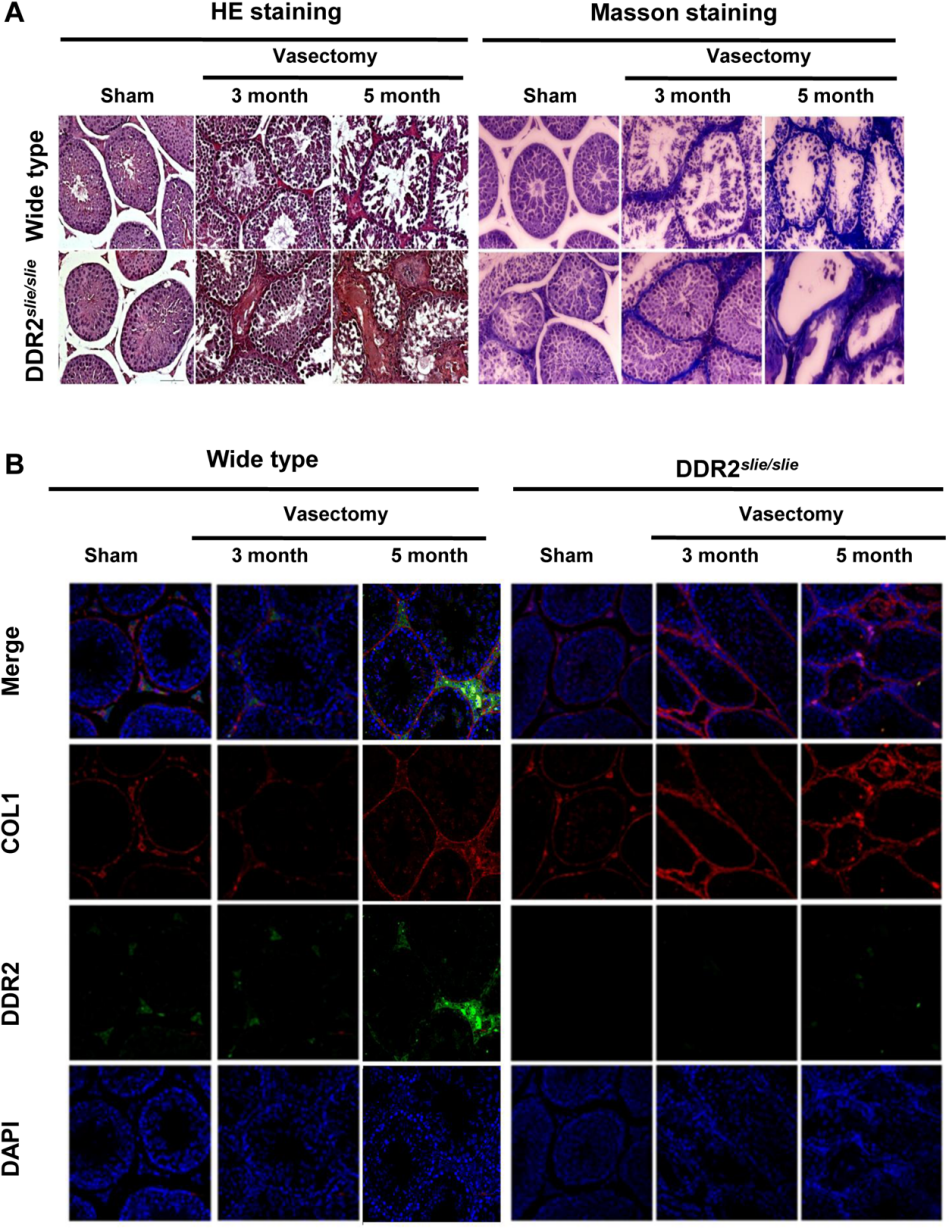
Establishment of murine vasectomy model and its effects on endogenous DDR2 and COL1 expression. **A** Establishment of murine vasectomy model was evaluated using Hematoxylin and eosin staining and Masson staining. Collagen fibers inside testicular interstitium displays blue coloration. **B** Immunolocalization of DDR2 and COL1 protein in testicular tissues from murine vasectomy model was carried out by immunofluorescence. Nuclear were demonstrated using DAPI staining.

Surgical removal or occlusion of a portion of vas deferens (vasectomy) is a unique long-term and reliable method of male contraception [22]. However, this procedure was developed for sterilization purposes and was not meant to be reversible [23]. Vasectomy induces testicular interstitial fibrosis in a time-dependent fashion and thereby inhibits spermatogenesis [23–26]. To further study the potential involvement of Ddr2 and COL1 in the pathogenesis of testicular interstitial fibrosis, we established the murine experimental vasectomy model in different experimental animals. In wild-type testis, interstitial accumulation of collagen and fibrosis formation were evident at Postoperative 5 month, whereas same pathological changes were notable as early as Postoperative 3 month in mutant testis (Fig 3A), indicating that the mutant testis may be more sensitive to the induction of testicular interstitial fibrosis. In line with the morphological analyses, immunofluorescence assay revealed a gradually increased expression of DDR2 and a relatively stable expression of COL1 in wild-type testis along the experimental period. In contrast, a significant increase of COL1 expression was evident in the testicular interstitial space of mutant mice from Postoperative 3 month onwards (Fig 3B).

### Steroidogenic capacity of mutant testis is more susceptible to collagen accumulation-induced damage

**Fig 4:**
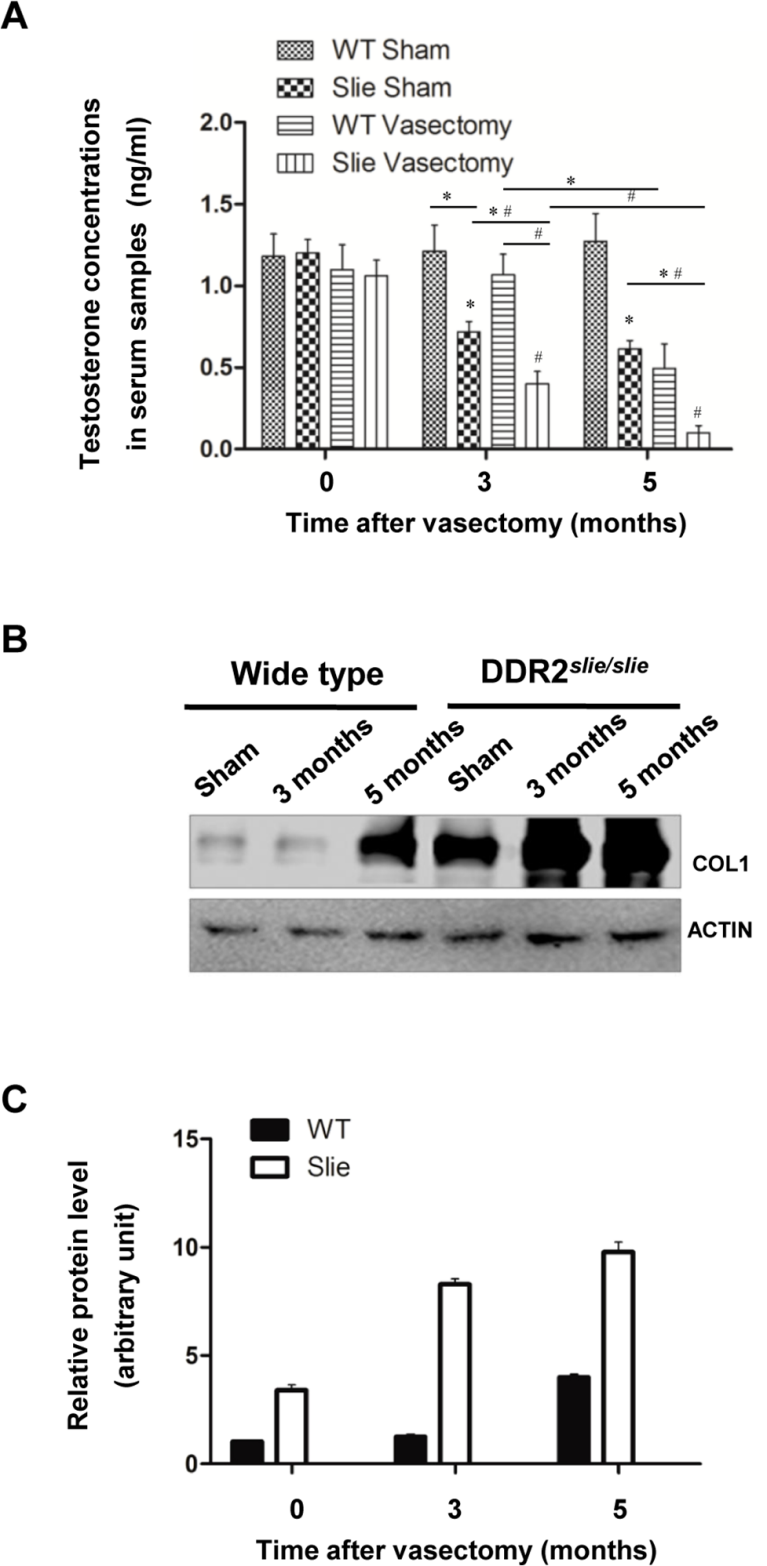
Effects of vasectomy on testicular COL1 expression and steroidogenesis. **A** The experimental murine vasectomy model was established as described in the **Materials and Methods** section. Animals were sacrificed at Postoperative 3 and 5 month, and serum testosterone (T) level was assessed by radioimmunoassay. Each data point represents the mean ± S.E.M. Bars with different letters in this panel indicate statistically significant differences in mean values from each other (*P* < 0.05), as determined by ANOVA and Tukey’s test. **B** Effects of vasectomy on testicular COL1 expression were determined using immunoblotting analyses in different experimental groups. Actin was served as internal control. **C** Densitometric scanning of immunoblots was performed in which the level of a target protein was normalized against the protein level in Group 0, which was arbitrarily set at 1.

Consistent with the data presented in Fig 3, vasectomy treatment significantly decreased testosterone levels in mutant testis from Postoperative 3 month onwards, while the same deleterious effect in wild-type testis could only be detectable from Postoperative 5 month. Of note, testicular steroidogenesis in mutant testis was substantially impaired from Postoperative 3 month even in the absence of vasectomy (Fig 4A). To further confirm the negative regulation of steroidogenesis by collagen accumulation, we evaluated the effect of vasectomy on COL1 expression profile by immunoblotting in different experimental groups. As expected, COL1 level in mutant testis was significantly higher than that of its corresponding wild-type controls (Fig 4B and 4C).

### Deregulated Ddr2 expression leads to impaired steroidogenesis in murine LCs

**Fig 5:**
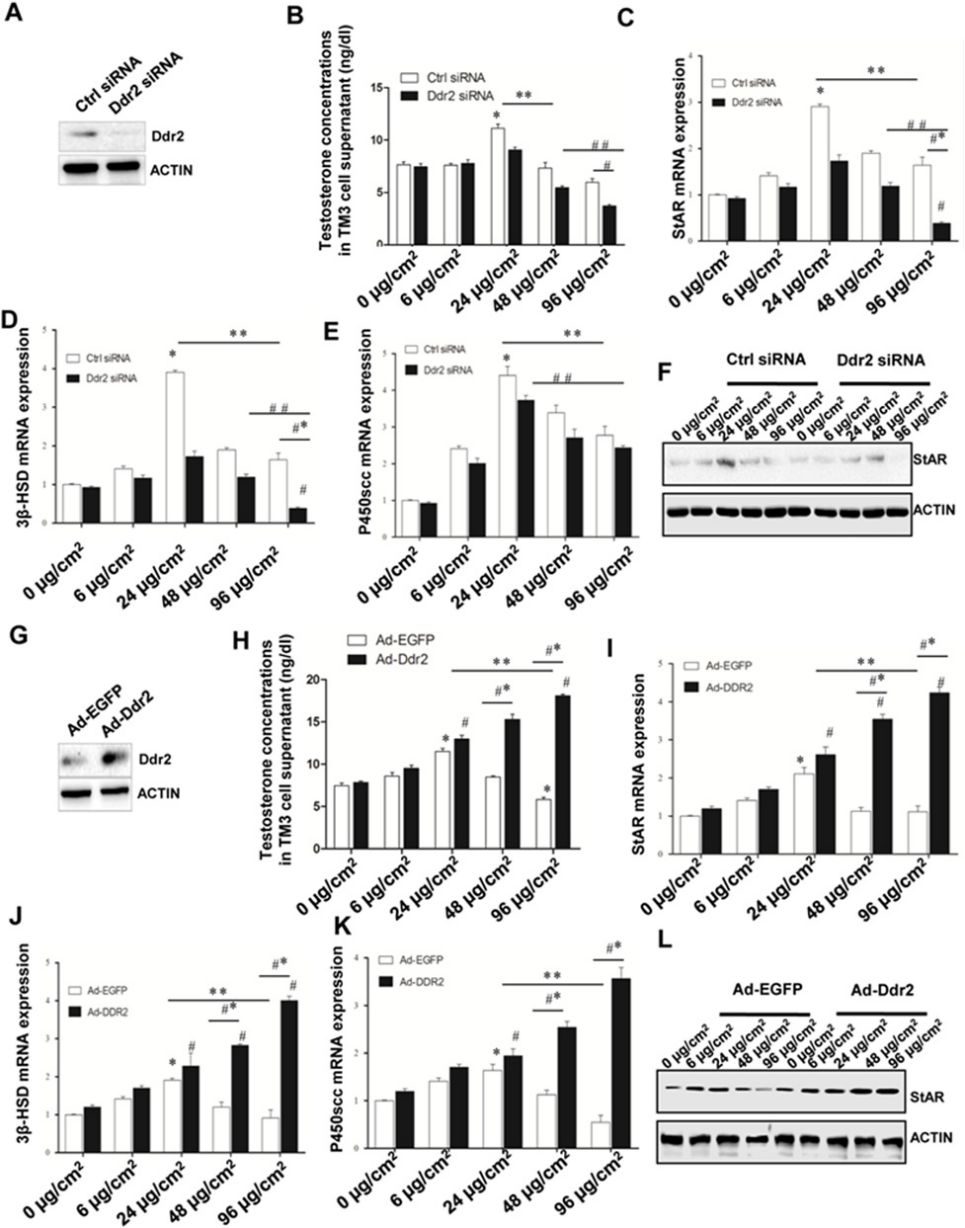
Effects of Ddr2 knockdown or Ddr2 overexpression on androgen production of TM3 Leydig cells in response to different concentrations of COL1 treatment. **A** Verification of Ddr2 knock down effect by siRNA treatment was performed using western blotting analyses. Actin was used as an internal control. **B** T concentrations in the culture medium of Ddr2-knockdown TM3 cells were determined using radioimmunoassay. **C-E** Expression levels of the testicular mRNAs encoding several key factors in the steroidogenic route were evaluated by real-time PCR. The data are expressed as the mean ± S.E.M. from three independent experiments. Parallel amplification of *Gapdh* was used as the internal control. *P < 0 .05 *vs* control siNC group. ^***#***^ *P* < 0 .05 *vs*. control siDDR2, **P < 0.05, 24 μg/cm^***2***^ siNC group *vs*. 96 μg/cm^***2***^ siNC group, ^***##***^ *P* < 0.05, siDDR2 24 μg/cm^***2***^ *vs*. siDDR2 96 μg/cm^***2***^, ^#^*P < 0.05, siNC 96μg/cm^***2***^ *vs*. siDDR2 96 μg/cm^***2***^. **F** Expression level of StAR in the presence of different concentrations of COL1 treatment was evaluated using western blotting analyses. **G** Verification of Ddr2 overexpression was performed using western blotting analyses. **H** T concentrations in the culture medium of Ddr2-overexpressing TM3 cells were determined using radioimmunoassay. **I-K** Expression levels of the testicular mRNAs encoding several key factors in the steroidogenic route were evaluated by real-time PCR. The data are expressed as the mean ± SEM from three independent experiments. Parallel amplification of *Gapdh* was used as the internal control. *P < 0 .05 *vs* control Ad-EGFP group. ^***#***^ *P* < 0 .05 *vs*. control Ad-DDR2, **P < 0.05, 24 μg/cm^***2***^ Ad-EGFP group *vs*. 96 μg/cm^***2***^ Ad-EGFP group, ^***##***^ *P* < 0.05, Ad-DDR2 24 μg/cm^***2***^ *vs*. Ad-DDR2 96 μg/cm^***2***^, ^#^*P < 0.05, Ad-EGFP 96μg/cm^***2***^ *vs*. Ad-DDR2 96 μg/cm^***2***^. **L** Expression level of StAR in the presence of different concentrations of COL1 treatment was evaluated using western blotting analyses. Immunolabeling of Actin is shown as loading control.

Next, we examined the effects of deregulation of Ddr2 expression on steroidogenesis of murine LCs. Since primary cultured Leydig cell will gradually lose their capacity of testosterone secretion, we used the TM3 cell lines, which response to LH stimulation and secrete testosterone, to evaluate the DDR2 function in steroidogenesis. Initially, we observed that ablation of endogenous Ddr2 can resulted in more than 5-fold increase in the COL1 expression (Fig 1B). 24 μg/cm^2^ has been shown to be the critical concentration which would affect LCs function [27]. In this context, we finally used 96 μg/cm^2^ as the highest concentration for COL1 treatment in TM3 cells to mimic the upregulation of endogenous COL1 in mutant testis. We firstly knocked down the endogenous Ddr2 expression using siRNA treatment (Fig 5A). Ablation of endogenous Ddr2 resulted in a significant decrease of T level in the culture medium of TM3 in the presence of higher concentration of COL1 treatment (Fig 5B). Consistently, levels of the mRNAs encoding several key factors in the steroidogenic route, such as steroid acute regulatory protein (*StAR*) (Fig 5C), 3*β* hydroxyl steroid dehydrogenase (*3β-HSD*) (Fig 5D), and the enzymes P450 side-chain cleavage (*P450scc*) (Fig 5E), were all significantly reduced in Ddr2-knockdown TM3 in the presence of higher concentration of COL1. Subsequent immunoblotting analyses revealed that ablation of Ddr2 impaired the expression levels of StAR in TM3 cells in response to higher concentration of COL1 treatment (Fig 5F). In TM3 overexpressing Ddr2, T concentration in the culture medium remained constantly stable along with the increase of COL1 elevation. However, when cells were cultured in highest concentration of COL1, we observed a significant decrease in the medium T level in control cells, a phenotype undetectable in TM3 overexpressing Ddr2 (Fig 5H). A similar change was also found in the expression levels of *StAR*, *3β-HSD* and *P450scc* (Fig 5I–5L). These data suggest that consumption of COL1 by Ddr2 may help to maintain a proper interstitial homeostasis for the testicular steroidogenesis.

### Disrupted expression of Ddr2 in human pathological testes correlates to the deregulated level of serum T

**Fig 6:**
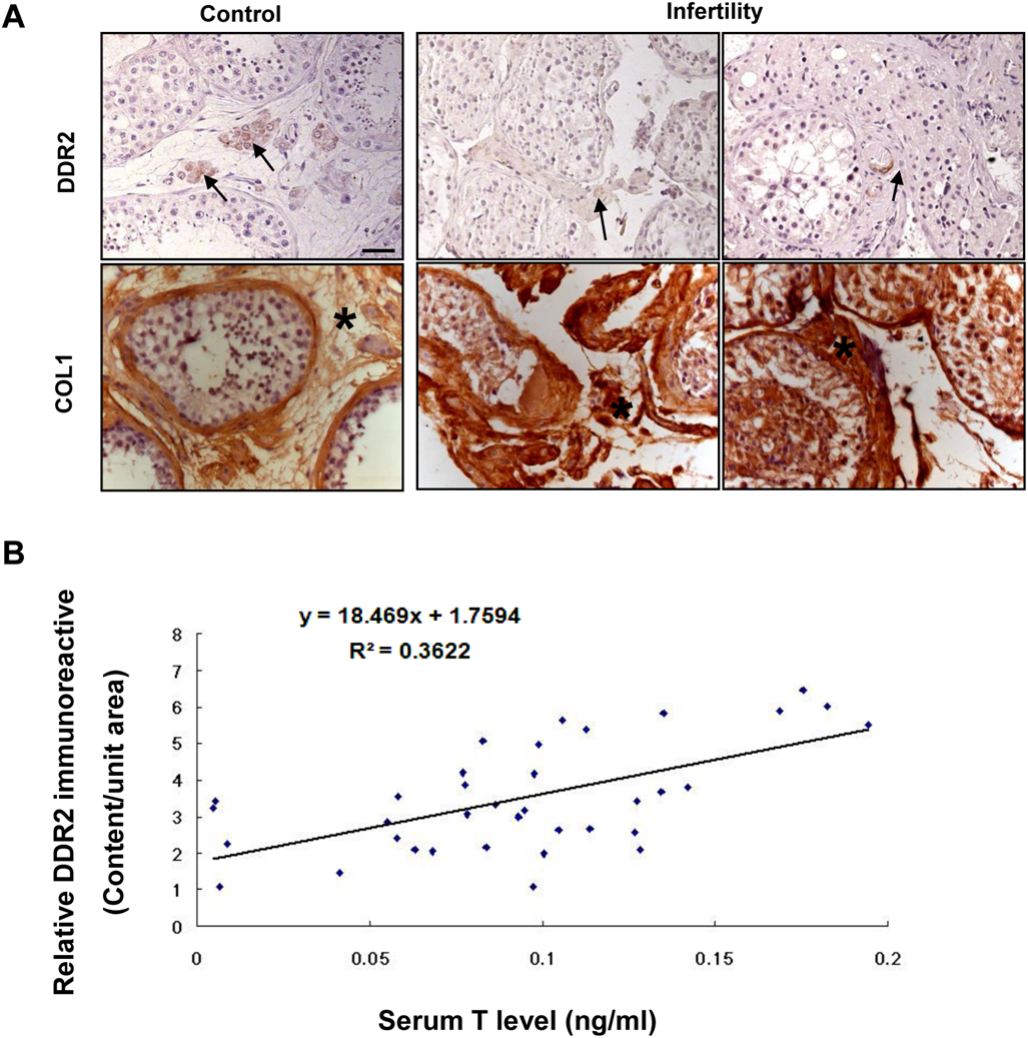
Deregulated testicular expression of Ddr2 positively correlates with serum T level in human infertile patients. **A** Immunohistochemical analysis of Ddr2 expression in human pathological testes. The arrows and asterisks denote the positive staining of Ddr2 in LCs and of COL1 in testicular interstitium, respectively. Bar = 25 μm. **B** correlation of relative Ddr2 immunoreactive content in human testis with serum T level was determined based on *Pearson*’s correlation coefficient with the aid of SPSS 15.0 software.

Deregulated expression of collagen has been frequently reported in the steriodogenic disruption of male infertile patients [28–31]. To this end, we next examined the localization of Ddr2 and COL1 immunoexpression in testicular tissues of patients with impaired spermatogenesis. Our analyses evidenced the dominant presence of both Ddr2 and COL1 immunostaining in the interstitial compartment. The intensity of Ddr2 immunoreactivity in the infertile testes was much lower than that in control testes. Conversely, COL1 immunostaining was significantly enhanced in the infertile testes (Fig 6A). In addition, we observed that the Ddr2 immunoreactivity in all groups correlated positively with the concentration of serum T (Fig 6B).

## Discussion

In the present study, evidence is provided for the potential involvement of Ddr2/COL1 cascade in the pathogenesis of male infertility in Ddr2^*slie/slie*^ mutant mice. The data comprise several main observations. First, COL1 is expressed in mouse testis throughout postnatal development, although the relative expression levels changed sharply along the study period: the highest expression values were detected in prepubertal-pubertal testicular samples, whereas the lowest levels were observed during the adult (70-d-old) period. Interestingly, this expression profile was negatively correlated to the expression pattern of Ddr2 along postnatal development, which showed the highest levels in the adult testis. Considering that Ddr2 is activated by collagen binding [32] and high concentration of collagen in the ECM usually has deleterious effects on the steroidogenic outcome of LCs [33], we hypothesize that high expression of Ddr2 consumes COL1 in the ECM, thus helping to maintain the low level of collagen concentration for the normal occurrence of steroidogenesis. The observation that COL1 is abnormally upregulated in Ddr2^*slie/slie*^ mutant testis also lends additional support to our hypothesis. Second, Fig 4A shows that the T levels were significantly reduced from Post-vasectomy 3 months onwards in mutant testis (Of note, we used 2-month old mice for the vasectomy experiments, so the mice at 3 months after surgery should be 5-month old). Two possibilities may account for this observation. First, this phenotype may reflect the LCs hypoplasia and hypotrophy at 5 months of age in Ddr2^*slie/slie*^ mutant testis. Alternatively, in wild type testis, expression of the cyclic AMP (cAMP) dependent protein kinase, which plays a pivotal role during testicular steroidogenesis, is much higher at 5 months of age than that at early adulthood. Type I Collagen has been reported to be able to regulate the activity of cAMP-dependent Protein Kinase A [34, 35]. So, it is very likely that the homeostasis between COL1 and cAMP-dependent Protein Kinase A is more susceptible to external factors-induced disruption at 5 months of age. In line with the above-mentioned phenotype, COL1 protein expression became significantly elevated in adult Ddr2^*slie/slie*^ mutant testis (Fig 1), and incubation with high concentration of COL1 significantly inhibited DDR2 expression in TM3 cells (Fig 5B–5K). Collectively, our present results strongly indicate that Ddr2 signaling may play a potential role in concert with COL1 in the maintenance of steroidogenic environment in adult Ddr2 ^*slie/slie*^ mutant male.

Vasectomy is a male contraceptive method involving only one small operation of vasal ligation. Emerging evidences demonstrate that spermatogenic damage may occur after vasectomy [36]. For example, it was previously reported that a larger proportion of fibrotic tissue blocks (between seminiferous tubules) were observed at the time of testicular biopsy in vasectomized men than in non-vasectomized men [24]. It is well documented that interstitial fibrogenesis can result in steroidogenic dysfunction in LCs [37]. To this end, we next used the experimental murine vasectomy model to further characterize the potential involvement of Ddr2/COL1 in the testicular fibrogenesis. Interestingly, the mutant testis appeared to be more vulnerable to the induction of testicular interstitial fibrosis by vasectomy. A tempting explanation is that the attenuated expression of Ddr2 may fail to consume the excess COL1, thus leading to the relative higher level of collagen accumulation inside testicular interstitium. More importantly, during the pathogenesis, Ddr2 ^*slie/slie*^ mutant mice showed a more prominent androgen deficiency than wild-type mice (Fig 4A). These results convincingly establish the potential involvement of Ddr2/COL1 cascade in the pathogenesis of vasectomy-induced androgen deficiency. It should be keep in mind that the phenotypes in the Ddr2 mutant may be mainly caused by loss of Ddr2 pathway, and the accumulation of the ligand COL1 may be a secondary effect due to the Ddr2 deficiency. Nevertheless, the modulatory role, if any, of Ddr2/COL1 signaling in the interstitial fibrogenesis, remains to be further elucidated.

Further evidence on the involvement of Ddr2/COL1 signaling in the steroidogenic homeostasis is provided by our studies using cultured TM3 cells. In this experimental setting, knock down of endogenous Ddr2 resulted in a significant decrease of circulated T level and of the mRNAs encoding several key factors in the steroidogenic route in TM3 in the presence of higher concentration of COL1, which was not observed in Ddr2-overexpressing cells. The canonical fibrogenesis parallels with necrosis or apoptosis of the adjacent somatic cells [38–40]. Therefore, as the essential component of the extracellular matrix, high dose of COL1 may have a direct “harmful” effect on TM3 function. Alternatively, it has been shown that COL4 mediated signaling is involved in the progenitor Leydig cells (PLCs) proliferation and is absent in the mature LCs [41]. In this sense, it is reasonable to propose that deregulated expression of COL1 may sabotage steroidogenic production, and Ddr2 activation may thereby operate as an important neutralizing mechanism to balance local COL1 concentration via its collagen binding activity. In favor of this hypothesis, we found that overexpression of Ddr2 helped the cells to maintain a normal steroidogenesis even in the presence of highest concentration of COL1. The mechanisms involved in such a rescuing response are presently under investigation. Of note, certain low concentration of COL1 treatment appeared to be also beneficial for T secretion of TM3 cells (Fig 5B). Therefore, when Ddr2 was knocked down, the gradual increase of COL1 level will stimulate steroidogenic production within certain time window. This may explain why the testosterone concentrations after knocking down *Ddr2* did not change as significantly as it changed after over-expressing *Ddr2* (Fig 5B vs. 5H). Taken together, the available data suggest that LCs steroidogenesis is subjected to a delicate control by the Ddr2/COL1 cascade.

The deteriorating effects of interstitial fibrogenesis have been frequently associated with androgen deficiency in many infertility-related diseases [42]. Our study demonstrated a dramatic reduce of Ddr2 expression in impaired testes as compared to that of the control testes. More importantly, Ddr2 expression correlated positively with concentration of serum T level, reinforcing the above-mentioned notion that Ddr2 signaling is required for the occurrence of normal steroidogenesis. Nevertheless, the relevance of the potential regulation between Ddr2 signaling in the presence of deregulated collagen expression in testicular interstitium is yet to be further defined. Worthy of note is that circulated T level is currently used as a most important fertility predictor for male patients, provided that the analysis was carried out with infertility duration [43]; this leaves unsolved the problem of evaluating spontaneous testicular steroidogenic damage during the very early phase in male infertility. To this end, our study also points to the diagnostic potential of Ddr2 signaling for this issue.

**Fig 7:**
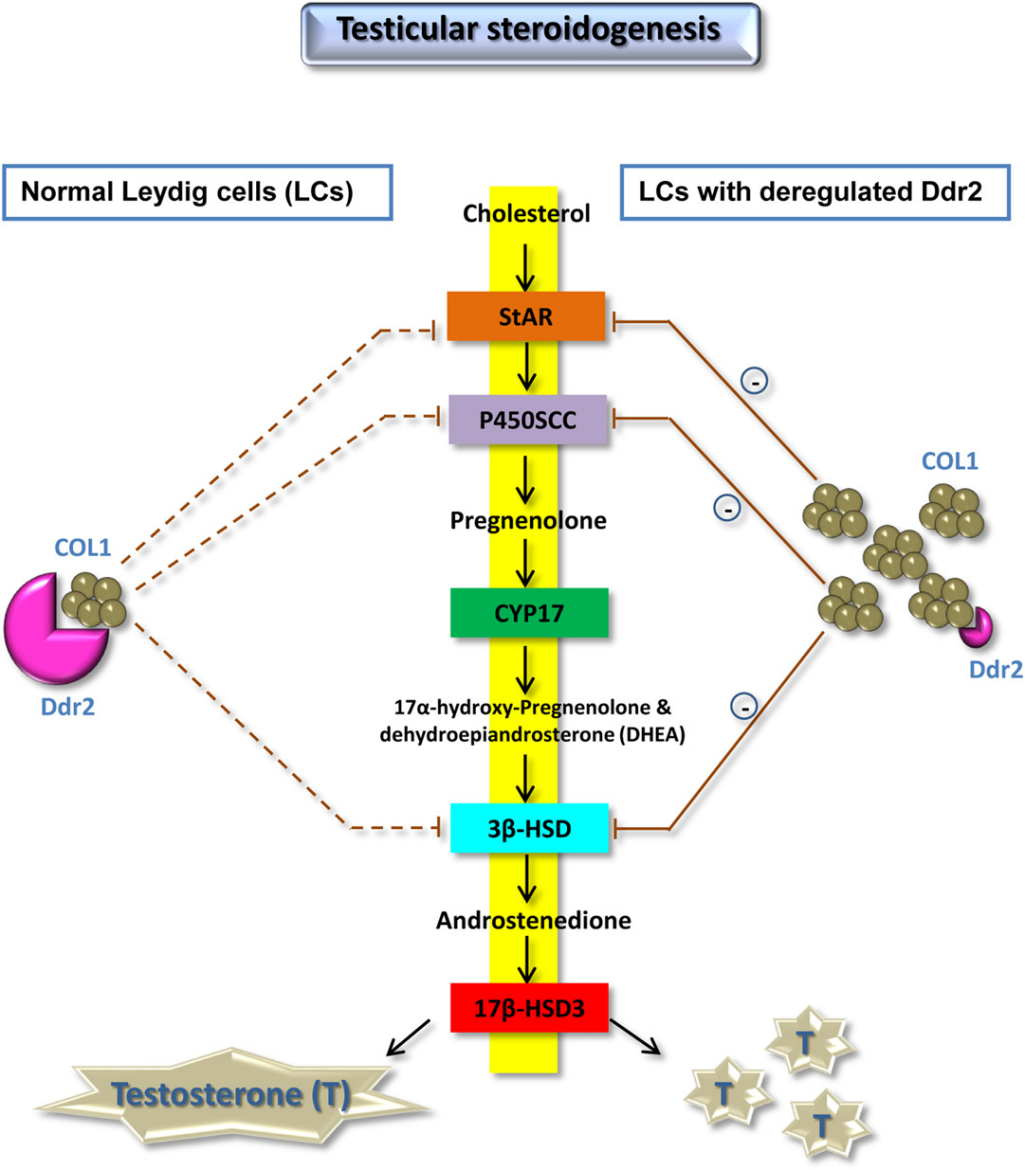
Summary diagram of the possible mechanisms related to deregulated Ddr2 expression contributing to the impairment of testicular steroidogenesis.

Components of ECM have been shown to alter Leydig cell steroidogenesis *in vitro*, substantiating the idea that Leydig cell steroidogenic activity and matrix environment are interdependent events [11]. Besides laminins, nidogens, and heparan sulfate proteoglycans, an ever-growing group of collagens and growth factors have been implicated in recent years in the control of testicular steroidogenic function [11]. Overall, the results presented herein suggest that Ddr2 participates in such a regulatory network in concert with COL1, thus providing an explanation for the reproductive phenotype of male *Ddr2* ^*slie/slie*^ mutant mice at the molecular level (Fig 7).

## References

[1] Khosravi R, Sodek KL, Faibish M, Trackman PC. Collagen advanced glycation inhibits its Discoidin Domain Receptor 2 (DDR2)-mediated induction of lysyl oxidase in osteoblasts. Bone. 2014;58:33–41. Epub 2013/10/15. doi: S8756-3282(13)00377-3 [pii] 10.1016/j.bone.2013.10.001 24120383 PMC3851576

[2] Olaso E, Ikeda K, Eng FJ, Xu L, Wang LH, Lin HC, et al DDR2 receptor promotes MMP-2-mediated proliferation and invasion by hepatic stellate cells. The Journal of clinical investigation. 2001;108(9):1369–78. 10.1172/JCI12373 11696582 PMC209436

[3] Vogel W, Gish GD, Alves F, Pawson T. The discoidin domain receptor tyrosine kinases are activated by collagen. Molecular cell. 1997;1(1):13–23. . 9659899 10.1016/s1097-2765(00)80003-9

[4] Ruiz PA, Jarai G. Collagen I induces discoidin domain receptor (DDR) 1 expression through DDR2 and a JAK2-ERK1/2-mediated mechanism in primary human lung fibroblasts. J Biol Chem. 2011;286(15):12912–23. Epub 2011/02/22. doi: M110.143693 [pii] 10.1074/jbc.M110.143693 21335558 PMC3075638

[5] Zhang Y, Su J, Yu J, Bu X, Ren T, Liu X, et al An essential role of discoidin domain receptor 2 (DDR2) in osteoblast differentiation and chondrocyte maturation via modulation of Runx2 activation. J Bone Miner Res. 2011;26(3):604–17. Epub 2010/08/25. 10.1002/jbmr.225 . 20734453

[6] Flynn LA, Blissett AR, Calomeni EP, Agarwal G. Inhibition of collagen fibrillogenesis by cells expressing soluble extracellular domains of DDR1 and DDR2. J Mol Biol. 2010;395(3):533–43. Epub 2009/11/11. doi: S0022-2836(09)01349-7 [pii] 10.1016/j.jmb.2009.10.073 19900459 PMC2813395

[7] Kano K, Marin de Evsikova C, Young J, Wnek C, Maddatu TP, Nishina PM, et al A novel dwarfism with gonadal dysfunction due to loss-of-function allele of the collagen receptor gene, Ddr2, in the mouse. Mol Endocrinol. 2008;22(8):1866–80. Epub 2008/05/17. doi: me.2007-0310 [pii] 10.1210/me.2007-0310 18483174 PMC2505327

[8] Kano K, Kitamura A, Matsuwaki T, Morimatsu M, Naito K. Discoidin domain receptor 2 (DDR2) is required for maintenance of spermatogenesis in male mice. Mol Reprod Dev. 2010;77(1):29–37. Epub 2009/08/15. 10.1002/mrd.21093 . 19681157

[9] Yu X, Hong S, Moreira EG, Faustman EM. Improving in vitro Sertoli cell/gonocyte co-culture model for assessing male reproductive toxicity: Lessons learned from comparisons of cytotoxicity versus genomic responses to phthalates. Toxicol Appl Pharmacol. 2009;239(3):325–36. Epub 2009/06/30. doi: S0041-008X(09)00263-4 [pii] 10.1016/j.taap.2009.06.014 19560483 PMC2735587

[10] Evaul K, Hammes SR. Cross-talk between G protein-coupled and epidermal growth factor receptors regulates gonadotropin-mediated steroidogenesis in Leydig cells. J Biol Chem. 2008;283(41):27525–33. Epub 2008/08/15. doi: M803867200 [pii] 10.1074/jbc.M803867200 18701461 PMC2562062

[11] Mazaud Guittot S, Verot A, Odet F, Chauvin MA, le Magueresse-Battistoni B. A comprehensive survey of the laminins and collagens type IV expressed in mouse Leydig cells and their regulation by LH/hCG. Reproduction. 2008;135(4):479–88. Epub 2008/03/28. doi: 135/4/479 [pii] 10.1530/REP-07-0561 . 18367508

[12] Odet F, Verot A, Le Magueresse-Battistoni B. The mouse testis is the source of various serine proteases and serine proteinase inhibitors (SERPINs): Serine proteases and SERPINs identified in Leydig cells are under gonadotropin regulation. Endocrinology. 2006;147(9):4374–83. Epub 2006/06/03. doi: en.2006-0484 [pii] 10.1210/en.2006-0484 . 16740973

[13] Lee NP, Cheng CY. Ectoplasmic specialization, a testis-specific cell-cell actin-based adherens junction type: is this a potential target for male contraceptive development? Hum Reprod Update. 2004;10(4):349–69. Epub 2004/06/12. 10.1093/humupd/dmh026 dmh026 [pii]. . 15192055

[14] Lui WY, Lee WM, Cheng CY. TGF-betas: their role in testicular function and Sertoli cell tight junction dynamics. Int J Androl. 2003;26(3):147–60. Epub 2003/05/21. doi: 410 [pii]. . 12755993 10.1046/j.1365-2605.2003.00410.x

[15] Diaz ES, Pellizzari E, Meroni S, Cigorraga S, Lustig L, Denduchis B. Effect of extracellular matrix proteins on in vitro testosterone production by rat Leydig cells. Mol Reprod Dev. 2002;61(4):493–503. Epub 2002/03/14. 10.1002/mrd.10111 [pii] 10.1002/mrd.10111. . 11891921

[16] Suarez-Quian CA, Martinez-Garcia F, Nistal M, Regadera J. Androgen receptor distribution in adult human testis. J Clin Endocrinol Metab. 1999;84(1):350–8. Epub 1999/01/27. 10.1210/jcem.84.1.5410 . 9920106

[17] Zhang S, Li W, Zhu C, Wang X, Li Z, Zhang J, et al Sertoli cell-specific expression of metastasis-associated protein 2 (MTA2) is required for transcriptional regulation of the follicle-stimulating hormone receptor (FSHR) gene during spermatogenesis. J Biol Chem. 2012;287(48):40471–83. Epub 2012/10/23. doi: M112.383802 [pii] 10.1074/jbc.M112.383802 23086931 PMC3504762

[18] Qu N, Terayama H, Naito M, Ogawa Y, Hirai S, Kitaoka M, et al Caput epididymitis but not orchitis was induced by vasectomy in a murine model of experimental autoimmune orchitis. Reproduction. 2008;135(6):859–66. Epub 2008/05/27. doi: 135/6/859 [pii] 10.1530/REP-08-0018 . 18502898

[19] Zhu CC, Zhang H, Zhang JS, Li Z, Zhao J, Li W, et al Inhibition of ghrelin signaling improves the reproductive phenotype of male ob/ob mouse. Fertil Steril. 2013;99(3):918–26. Epub 2012/12/13. doi: S0015-0282(12)02434-X [pii] 10.1016/j.fertnstert.2012.11.022 . 23232360

[20] Zhang S, Zeng Y, Qu J, Luo Y, Wang X, Li W. Endogenous EGF maintains Sertoli germ cell anchoring junction integrity and is required for early recovery from acute testicular ischemia/reperfusion injury. Reproduction. 2013;145(2):177–89. Epub 2012/12/18. doi: REP-12-0336 [pii] 10.1530/REP-12-0336 . 23241343

[21] Li W, Zeng Y, Zhao J, Zhu CJ, Hou WG, Zhang S. Upregulation and nuclear translocation of testicular ghrelin protects differentiating spermatogonia from ionizing radiation injury. Cell Death Dis. 2014;5:e1248. Epub 2014/05/24. doi: cddis2014223 [pii] 10.1038/cddis.2014.223 24853426 PMC4047875

[22] Belleannee C, Legare C, Calvo E, Thimon V, Sullivan R. microRNA signature is altered in both human epididymis and seminal microvesicles following vasectomy. Human reproduction. 2013;28(6):1455–67. 10.1093/humrep/det088 . 23539611

[23] Shiraishi K, Takihara H, Naito K. Influence of interstitial fibrosis on spermatogenesis after vasectomy and vasovasostomy. Contraception. 2002;65(3):245–9. . 11929647 10.1016/s0010-7824(01)00311-0

[24] Xiang Y, Luo P, Cao Y, Yang ZW. Long-term effect of vasectomy on spermatogenesis in men: a morphometric study. Asian J Androl. 2013;15(3):434–6. Epub 2013/02/26. doi: aja2012154 [pii] 10.1038/aja.2012.154 23435469 PMC3739648

[25] Shiraishi K, Takihara H, Naito K. Quantitative analysis of testicular interstitial fibrosis after vasectomy in humans. Aktuelle Urol. 2003;34(4):262–4. Epub 2003/10/21. 10.1055/s-2003-41612 . 14566680

[26] Jarow JP, Goluboff ET, Chang TS, Marshall FF. Relationship between antisperm antibodies and testicular histologic changes in humans after vasectomy. Urology. 1994;43(4):521–4. Epub 1994/04/01. . 8154074 10.1016/0090-4295(94)90246-1

[27] Diaz ES, Pellizzari E, Casanova M, Cigorraga SB, Denduchis B. Type IV collagen induces down-regulation of steroidogenic response to gonadotropins in adult rat Leydig cells involving mitogen-activated protein kinase. Mol Reprod Dev. 2005;72(2):208–15. Epub 2005/07/23. 10.1002/mrd.20259 . 16037942

[28] Raleigh D, O'Donnell L, Southwick GJ, de Kretser DM, McLachlan RI. Stereological analysis of the human testis after vasectomy indicates impairment of spermatogenic efficiency with increasing obstructive interval. Fertil Steril. 2004;81(6):1595–603. Epub 2004/06/15. 10.1016/j.fertnstert.2003.10.046 S0015028204002560 [pii]. . 15193483

[29] Fagundes AK, Oliveira EC, Tenorio BM, Melo CC, Nery LT, Santos FA, et al Injection of a chemical castration agent, zinc gluconate, into the testes of cats results in the impairment of spermatogenesis: a potentially irreversible contraceptive approach for this species? Theriogenology. 2014;81(2):230–6. Epub 2013/11/19. doi: S0093-691X(13)00376-2 [pii] 10.1016/j.theriogenology.2013.09.013 . 24238399

[30] Kumar PA, Pitteloud N, Andrews PA, Dwyer A, Hayes F, Crowley WF Jr., et al Testis morphology in patients with idiopathic hypogonadotropic hypogonadism. Hum Reprod. 2006;21(4):1033–40. Epub 2006/01/07. doi: dei444 [pii] 10.1093/humrep/dei444 . 16396935

[31] Gulkesen KH, Erdogru T, Sargin CF, Karpuzoglu G. Expression of extracellular matrix proteins and vimentin in testes of azoospermic man: an immunohistochemical and morphometric study. Asian J Androl. 2002;4(1):55–60. Epub 2002/03/22. . 11907629

[32] Yan Z, Jin S, Wei Z, Huilian H, Zhanhai Y, Yue T, et al Discoidin domain receptor 2 facilitates prostate cancer bone metastasis via regulating parathyroid hormone-related protein. Biochim Biophys Acta. 2014;1842(9):1350–63. Epub 2014/05/03. doi: S0925-4439(14)00105-7 [pii] 10.1016/j.bbadis.2014.04.018 . 24787381

[33] Erdogru T, Gulkesen KH, Bahceci M, Karpuzoglu G, Baykara M. The role of expression of extracellular matrix proteins and epidermal growth factor receptor activity on fertilization capacity of testicular harvested spermatozoa. Andrologia. 2002;34(2):98–106. Epub 2002/04/23. doi: 482 [pii]. . 11966576 10.1046/j.0303-4569.2001.00482.x

[34] Margarucci L, Roest M, Preisinger C, Bleijerveld OB, van Holten TC, Heck AJ, et al Collagen stimulation of platelets induces a rapid spatial response of cAMP and cGMP signaling scaffolds. Mol Biosyst. 2011;7(7):2311–9. Epub 2011/05/21. 10.1039/c1mb05145h . 21597619

[35] Yan R, Wang Z, Yuan Y, Cheng H, Dai K. Role of cAMP-dependent protein kinase in the regulation of platelet procoagulant activity. Arch Biochem Biophys. 2009;485(1):41–8. Epub 2009/03/10. doi: S0003-9861(09)00061-7 [pii] 10.1016/j.abb.2009.02.014 . 19268649

[36] Cook LA, Van Vliet HA, Lopez LM, Pun A, Gallo MF. Vasectomy occlusion techniques for male sterilization. Cochrane Database Syst Rev. 2014;3:CD003991 Epub 2014/04/01. 10.1002/14651858.CD003991.pub4 . 24683020 PMC7173716

[37] Svechnikov K, Izzo G, Landreh L, Weisser J, Soder O. Endocrine disruptors and Leydig cell function. J Biomed Biotechnol. 2010;2010 Epub 2010/09/24. 10.1155/2010/684504 20862379 PMC2938463

[38] Zhou L, Li Y, Zhou D, Tan RJ, Liu Y. Loss of Klotho contributes to kidney injury by derepression of Wnt/beta-catenin signaling. J Am Soc Nephrol. 2013;24(5):771–85. Epub 2013/04/06. doi: ASN.2012080865 [pii] 10.1681/ASN.2012080865 23559584 PMC3636797

[39] Biernacka A, Dobaczewski M, Frangogiannis NG. TGF-beta signaling in fibrosis. Growth Factors. 2011;29(5):196–202. Epub 2011/07/12. 10.3109/08977194.2011.595714 . 21740331 PMC4408550

[40] Cheng JH, She H, Han YP, Wang J, Xiong S, Asahina K, et al Wnt antagonism inhibits hepatic stellate cell activation and liver fibrosis. Am J Physiol Gastrointest Liver Physiol. 2008;294(1):G39–49. Epub 2007/11/17. doi: 00263.2007 [pii] 10.1152/ajpgi.00263.2007 . 18006602

[41] Anbalagan M, Rao AJ. Collagen IV-mediated signalling is involved in progenitor Leydig cell proliferation. Reprod Biomed Online. 2004;9(4):391–403. Epub 2004/10/30. . 15511338 10.1016/s1472-6483(10)61274-6

[42] Mountain DJ, Freeman BM, Kirkpatrick SS, Beddies JW, Arnold JD, Freeman MB, et al Androgens regulate MMPs and the cellular processes of intimal hyperplasia. J Surg Res. 2013;184(1):619–27. Epub 2013/06/19. doi: S0022-4804(13)00554-4 [pii] 10.1016/j.jss.2013.05.070 . 23773714

[43] Guo SJ, Sun ZJ, Li W. New insights about the early diagnosis of fertility impairment in varicoceles: the DNA repair gene example. Med Hypotheses. 2012;78(4):536–8. Epub 2012/02/07. doi: S0306-9877(12)00040-0 [pii] 10.1016/j.mehy.2012.01.029 . 22305334

